# Links between the rumen microbiota, methane emissions and feed efficiency of finishing steers offered dietary lipid and nitrate supplementation

**DOI:** 10.1371/journal.pone.0231759

**Published:** 2020-04-24

**Authors:** Jenna M. Bowen, Paul Cormican, Susan J. Lister, Matthew S. McCabe, Carol-Anne Duthie, Rainer Roehe, Richard J. Dewhurst

**Affiliations:** 1 Animal & Grassland Research and Innovation Centre, Teagasc, Grange, Dunsany, Co. Meath, Ireland; 2 IBERS, Aberystwyth University, Aberystwyth, Wales, United Kingdom; 3 SRUC, Edinburgh, Scotland, United Kingdom; The University of Sydney, AUSTRALIA

## Abstract

Ruminant methane production is a significant energy loss to the animal and major contributor to global greenhouse gas emissions. However, it also seems necessary for effective rumen function, so studies of anti-methanogenic treatments must also consider implications for feed efficiency. Between-animal variation in feed efficiency represents an alternative approach to reducing overall methane emissions intensity. Here we assess the effects of dietary additives designed to reduce methane emissions on the rumen microbiota, and explore relationships with feed efficiency within dietary treatment groups. Seventy-nine finishing steers were offered one of four diets (a forage/concentrate mixture supplemented with nitrate (NIT), lipid (MDDG) or a combination (COMB) compared to the control (CTL)). Rumen fluid samples were collected at the end of a 56 d feed efficiency measurement period. DNA was extracted, multiplexed 16s rRNA libraries sequenced (Illumina MiSeq) and taxonomic profiles were generated. The effect of dietary treatments and feed efficiency (within treatment groups) was conducted both overall (using non-metric multidimensional scaling (NMDS) and diversity indexes) and for individual taxa. Diet affected overall microbial populations but no overall difference in beta-diversity was observed. The relative abundance of Methanobacteriales (*Methanobrevibacter* and *Methanosphaera*) increased in MDDG relative to CTL, whilst *VadinCA11* (Methanomassiliicoccales) was decreased. Trimethylamine precursors from rapeseed meal (only present in CTL) probably explain the differences in relative abundance of Methanomassiliicoccales. There were no differences in Shannon indexes between nominal low or high feed efficiency groups (expressed as feed conversion ratio or residual feed intake) within treatment groups. Relationships between the relative abundance of individual taxa and feed efficiency measures were observed, but were not consistent across dietary treatments.

## Introduction

Ruminant production systems are a significant source of greenhouse gas (GHG) emissions worldwide, with enteric methane accounting for approximately 10–12% of global methane emissions [1,2]. Because a negative relationship has previously been reported between feed efficiency and methane emissions in beef cattle [3], it is important also to consider potential effects of anti-methanogenic treatments on feed efficiency. Diet has previously been the basis of effective mitigations of methane emissions [4], but we need also to consider GHG mitigation through improving feed efficiency.

Next-generation sequencing (NGS) has enabled more rapid and detailed descriptions of the microbiota and recent studies have shown that even small shifts are associated with productivity [5, 6]. Most studies have focused on diet effects [7–9], with fewer looking at relationships with feed efficiency [5, 6, 10, 11]. Ross et al. [12] showed clear differences in the microbiota from animals offered the same diet, whilst Roehe et al. [13] identified some of the mechanisms involved in host control of the rumen microbiome. There is no evidence about the potential role of the rumen microbiota in between-animal variation in feed efficiency in a natural population of animals (i.e. not selecting for extremes in efficiency), though the contribution of rumen fermentation to digestion of fibrous feeds has led to an assumption that digestion is important.

The effects of dietary nitrate, lipid and combinations of nitrate and lipid on feed efficiency and methane emissions have been previously assessed [14–16]. However, there remains uncertainty about the mechanisms and interactions involved. Many rumen methanogenic Archaea produce methane by converting H_2_ and CO_2_ to methane in a seven step pathway [17]. There has been recent interest in another group of Archaea, Thermoplasmata (reclassified as Methanomassiliicoccales [18]) which utilize methanol and methylamines in the production of methane (‘methylotrophic’ as opposed to ‘hydrogenotrophic’ methanogenesis). Poulsen et al. [19] showed a reduction in methane emissions due to rapeseed oil supplementation. These authors identified the methanogenic properties of Thermoplasmata due to the presence of methylcoenzyme M reductase genes and showed that Thermoplasmata produced more methane (when expressed as CH_4_-to-CO_2_ ratio) than the hydrogenotrophs (Methanobacteriales).

The aims of the present study were to assess the effects of dietary additives on the rumen microbiota and discover potential relationships with methane production and feed efficiency. In contrast to other studies [5, 6], our work has explored the full range of variation in feed efficiency within groups of animals offered the same diets, rather than selecting extreme high- and low-feed efficiency animals.

## Materials and methods

### Animal study and sample collection

This experiment was conducted at Scotland’s Rural College (SRUC) Beef and Sheep Research Centre. The experimental protocol was approved by SRUC’s Animal Welfare and Ethical Review Body and was conducted in accordance with the requirements of the UK Animals (Scientific Procedures) Act, 1986. The overall study design and details of animals used in this study have been previously described by Duthie et al. [14]. In brief, crossbred Aberdeen Angus (AAx) and Limousin (LIMx) animals were used. Steers were offered a basal diet consisting of forage to concentrate ratio of 550:450 (g/kg DM)–full dietary contents can be found in Table 1. In addition to the basal diet, steers were offered one of four treatments; (i) control (CTL; n = 20) containing rapeseed meal as the main protein source, (ii) nitrate (NIT; n = 20) supplemented in the form of calcium nitrate, (iii) lipid (MDDG; n = 20) supplemented in the form of maize distillers dark grains replacing rapeseed meal (which increased dietary ether extract from 24.0 to 36.7 g/kg DM), or (iv) a combination of NIT and MDDG diets (COMB; n = 19) which increased dietary ether extract from 24.0 to 35.9 g/kg DM. Steers were offered fresh forage daily and had continuous access to both feed and fresh water throughout the trial. After a 4 week adaptation period to dietary treatments, steers underwent a 56-day performance test, during which feed intake and growth were recorded. Feed conversion ratio (FCR) and residual feed intake (RFI) were calculated for each animal. Greenhouse gas emissions were measured over a period of 12 weeks after the performance test (n = 72), as described by Duthie et al. [14]. Rumen samples were collected at the end of the performance test period immediately after fresh feed was offered. Rumen samples were collected by inserting a stomach tube nasally (16 x 2700 mm Equivet Stomach Tube, JørgenKruuse A/S, Langeskov, Denmark) and aspirating manually. Rumen liquor was passed through 4 layers of cheesecloth and 5 ml mixed with 10 ml phosphate buffered saline containing glycerol (30% v/v). Samples were immediately stored on ice and transferred to -20°C within 3 h of collection.

**Table 1:** Ingredient composition for each of the 4 experimental diets (g/kg DM)–Duthie et al. (2018) [14].

Ingredient	CTL	MDDG	NIT	COMB
Grass silage	210	209	211	210
Whole-crop barley silage	347	346	347	346
Barley	336	289	388	263
Rapeseed meal	79	0	0	0
Calcinit	0	0	25	25
Maize distillers grains	0	128	0	127
Molasses	19	19	20	19
Vitamins and minerals	9	9	9	9

### DNA extraction

Rumen samples were crushed to a fine powder in liquid nitrogen using a pestle and mortar before being transferred to a -80°C freezer. One milliliter of lysis buffer was added to a 600 mg rumen sample (thawed). DNA was extracted using the repeated beat beating and column filtration method [20]. DNA quality was assessed on an agarose gel, and quantified using Nanodrop 1000 (ThermoFisher Scientific, Dublin, Ireland).

### Library preparation and next generation sequencing

Libraries were prepared by PCR amplification of the hypervariable (V4) region of the 16S rRNA gene. PCR amplification was carried out using barcoded 16S Illumina primers containing 12 bp barcodes (515F/806R rcbc; [21, 22]; full primer details can be found in S1 Table), Q5 Hot Start-High Fidelity DNA Polymerase and High GC Content Enhancer (New England Biolabs Inc., Massachusetts, USA). Cycle conditions were 94°C (2 min), followed by 30 cycles of 94°C (10 s), 68°C (20 s) and 72°C (1 min). Libraries were immediately purified using the QIAquick PCR Purification Kit (Qiagen, Manchester, UK) and quantified using a Nanodrop 1000 (ThermoFisher Scientific, Dublin, Ireland). Each sample was combined into one of two pools in equimolar concentrations; NIT/COMB (n = 39) and MDDG/CTL (n = 40). Each pool was gel purified using the QIAquick Gel Extraction Kit (Qiagen, Manchester, UK), and checked for size with a DNA1000 chip on an Agilent 2100 Bioanalyser (Agilent Technologies, Cork, Ireland). The pooled libraries were then quantified by qPCR on an ABI7500 FAST real time qPCR machine (Life Technologies, Renfrew, UK) using the Universal qPCR master mix from the Kapa library quantification kit for Illumina platforms (Kapa Biosystems, United States). Pooled libraries were then diluted to 2 nM, denatured with sodium hydroxide, spiked with denatured PhiX version 3 library (Illumina, San Diego, USA) (6:4 volume:volume, Pooled libraries:PhiX V3 library) and loaded into a 300 cycle version 2 MiSeq reagent cartridge (Illumina) which was run on an Illumina MiSeq.

### Sequencing data clean-up

Raw sequence reads were quality controlled using the BBduk (https://sourceforge.net/projects/bbmap/) Java package. This was used to trim low quality bases (<20 Phred score) from the 3′ end of sequence read pairs, remove adaptor contamination and remove read pairs containing ambiguous bases. Read pairs with an insert size (length of template molecule) shorter than the sum of the lengths of read 1 and read 2 were merged into a single, longer read. Size selection of 253 bp ±20 bp sequences was performed with an in-house Perl script. Chimeric sequences were identified using usearch61 against the GreenGenes database (v13.5; http://greengenes.lbl.gov) and removed. OTUs were assembled using the open reference method (a combination of reference based and *de novo* methodologies) using usearch61 with a 97% similarity used to cluster reads into individual OTUs (QIIME1 v1.9; [23]). Taxonomy was assigned to these OTUs using the RDP classifier (v2.2) and the GreenGenes database. Associated sequence files have been submitted to NCBI Sequence Read Archive (Accession no. PRJNA402989). Unassigned taxa (unassigned at any level) were removed. Abundance estimates were calculated by summing read counts of OTUs with identical taxonomic assignments from Kingdom to Genus taxonomic level (S1 Table). Samples were assigned to four groups based on dietary treatment: (i) CTL, (ii) MDDG, (iii) NIT, and (iv) COMB. Samples were rarefied to the lowest read number (~22,600) across all samples with Shannon diversities (H) calculated at each iteration.

### Statistical analysis

Steers were ranked by RFI and assigned to either a low or high RFI group (balancing for breed) within each dietary treatment (CTL, NIT and MDDG; low, n = 10, high n = 10; COMB low, n = 9, high, n = 10; S1 Fig). Note that the single high RFI value was not due to underlying health issues. The same process was carried out using FCR. These new factors were termed RFI Level and FCR Level respectively.

Non-metric dimensional scaling (NMDS) ordination plots were created using the metaMDS() function in the VEGAN package (Community Ecology Package, V 2.5–2) of R Studio (V 3.4.3), in which OTUs were rarefied to the lowest sequence number. In order to assess the effects of dietary treatment, nominal RFI level and nominal FCR level on microbial populations, permutational multivariate analysis of variance (PERMANOVA) was carried out using the adonis() function in VEGAN. Beta-diversity between all groups (i.e. dietary treatments) was assessed using the betadisper() function in VEGAN. Differences in phyla between diets were assessed using a Pairwise Wilcox Test in RStudio using the pairwise.wilcox.test() function, a threshold of P < 0.05) was set.

Taxa which had 0.00% relative abundances across any individual diet (at genus level) were removed to produce a core microbiota across all diets. Firmicutes to Bacteroidetes ratio (F:B) was calculated from corresponding relative abundances. Pearson correlations were examined between FCR and RFI values and Firmicutes:Bacteroidetes (F:B) ratio within each treatment group.

Diet effects on relative abundances of genera were estimated using the Kruskal-Wallis (non-parametric) test in the STAMP statistical package (STAMP; [24]) with a Benjamini-Hochberg false discovery rate applied. Values for each of the treatment groups (NIT; MDDG; COMB) were subsequently compared to CTL diet across all samples. Subsequently, analysis was repeated on samples with associated methane measurements, particular interest was taken in taxa associated with methane emissions. Values with P < 0.05 were classed as significant.

Shannon diversity indexes were calculated in QIIME1 for each of the samples to assess both species evenness and richness, data was visualised using box plots. Differences between dietary treatments were assessed using analysis of variance (R Studio). Differences in Shannon diversity indexes within each diet were assessed using general linear regression (REML; GenStat), with nominal feed efficiency level as the fixed effect. Relative abundances that averaged < 1% across each of the diets were removed at both phylum and family level as described by McCann et al. [5]. The effect of taxonomic group (phylum and family level) on feed efficiency, whether expressed as nominal groups or individual values, was assessed using General Linear Regression for each treatment group separately.

## Results

### Animal measurements

Full details of performance data and emissions can be found in Duthie et al. [14]. In brief, dietary treatments containing nitrate increased FCR (reduced feed efficiency; P < 0.05), but not RFI. Treatments containing lipid did not (P > 0.05) influence feed efficiency (whether expressed as FCR or RFI). AAx steers had higher ADG (P < 0.01) and higher DMI (P < 0.001) than LIMx steers and were less feed efficient (RFI; P < 0.01). There were no interactions (P > 0.05) between breed and dietary treatment for performance measures. There were no significant differences in dry matter intakes between dietary treatments, whether expressed as kg/day or g/kg body weight. Inclusion of nitrate resulted in decreased production of methane relative to CTL whether expressed on an absolute grams per day (27 g/d/11% reduction in NIT, 36 g/d/15% reduction in COMB; P < 0.001) or grams per kg dry matter intake basis (1.9 g/kg DMI/8% reduction in NIT, 3.1 g/kg DMI/13% reduction in COMB; P < 0.001). The inclusion of lipid resulted in a numerical reduction in methane emissions on a grams per day (8 g/d/3% reduction in MDDG, 36 g/d/15% reduction in COMB) and DMI basis (0.6 g/kg DMI/3% reduction in MDDG, 3.1 g/kg DMI/13% reduction in COMB) however this was non-significant (P > 0.05). There were no significant interactions between the inclusion of lipid and nitrate (P >0.05).

### Sequence data

**Fig 1:**
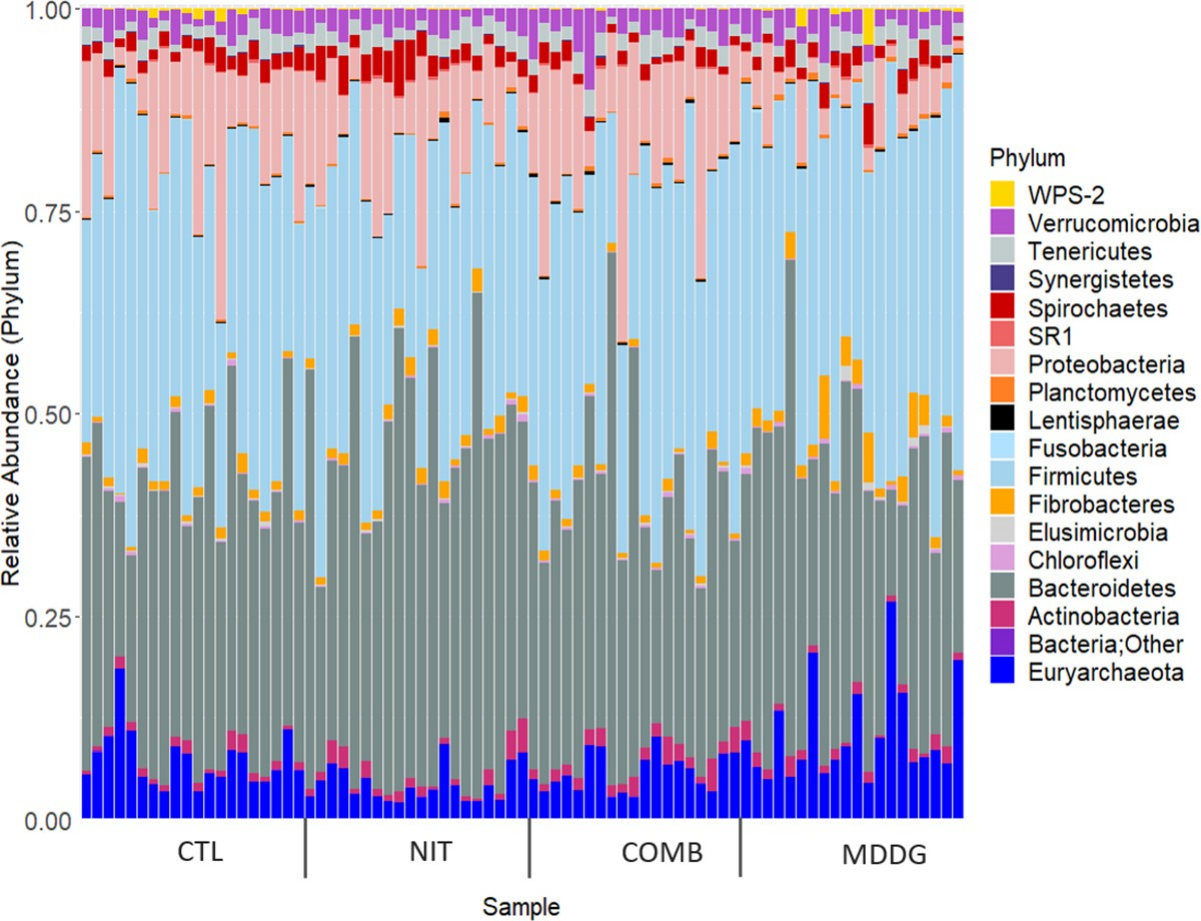
Taxa summary plot across the 4 dietary treatments (CTL, NIT, COMB, MDDG).

Overall, 6,667,019 reads were generated which reduced to an average (± standard deviation) of 82,033 (±36,021) reads per sample after filtering. Full details of taxa for each of the dietary treatments can be found in S1 Table. Rarefaction analysis confirmed that sequencing was performed to a sufficient depth–see S2 Fig. Eighteen phyla were recorded once unclassified taxa were removed. Details of the most abundant phyla are provided in Table 2: Firmicutes, Bacteroidetes, Proteobacteria, Euryarchaeota, Verrucomicrobia, Spirochaetes, Tenericutes, Fibrobacteres and Actinobacteria were all present at relative abundances > 1%. Fig 1 shows differences in the relative abundance of phyla across dietary treatments.

**Table 2:** Mean relative abundance (%) and standard error of phyla for the rumen microbiota from the 4 dietary treatments (CTL, MDDG, NIT, and COMB).

Taxonomy (Phylum)	CTL	NIT	COMB	MDDG	Standard error mean (P-Value)
	Mean Rel Ab. (%)	Mean Rel Ab. (%)	Mean Rel Ab. (%)	Mean Rel Ab. (%)	
Firmicutes	36.7^ab^	31.5^b^	35.4^ab^	38.4^a^	1.001 (0.088)
Bacteroidetes	34.0^b^	42.0^a^	33.5^b^	33.7^b^	1.169 (0.021)
Proteobacteria	12.0^a^	10.7^a^	13.8^a^	4.4^b^	0.805 (<0.001)
Euryarchaeota	7.3^ab^	4.3^c^	5.6^b^	10.5^a^	0.503 (<0.001)
Verrucomicrobia	2.4	2.4	3.4	2.7	0.163 (0.619)
Spirochaetes	1.9^ab^	2.8^a^	1.6^b^	2.0^ab^	0.126 (0.025)
Tenericutes	1.9^b^	2.3^a^	2.2^ab^	2.3^ab^	0.100 (0.215)
Fibrobacteres	1.1^bc^	1.5^ab^	1.1^c^	2.6^a^	0.140 (<0.001)
Actinobacteria	1.1^b^	1.4^b^	1.8^a^	1.3^b^	0.095 (0.013)

Different letters within column are significantly different at P < 0.05.

One hundred and eighty one genera were recorded; *Prevotella* predominated, with 25.0%, 25.4%, 32.4% and 24.1% relative abundance in samples from CTL, MDDG, NIT and COMB diets respectively. Other taxa present at relative abundances > 5% include genera *Ruminococcus* and *Methanobrevibacter*, families Succinivibrionaceae, Methanobacteriaceae and Ruminococcaceae and the order Clostridiales. Of the 181 genera identified, 164 were present in samples from all diets and so were identified as the core microbiota (S2 Table). The remaining 17 taxa were unique to at least one of the dietary treatments; no taxa were unique to a single diet.

### Diet effects on microbial communities

**Fig 2:**
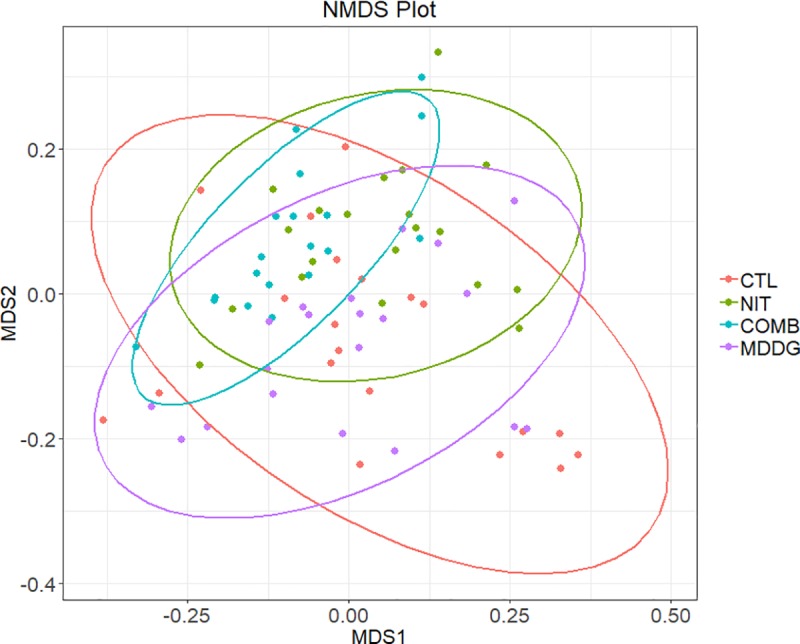
Difference in community structure between dietary treatments using NMDS plot showing the 95% confidence interval ellipse for each dietary treatment.

Overall differences in community structure were assessed using NMDS (Fig 2). Dietary treatment had a significant effect on microbial populations (R^2^ = 0.141, P < 0.001). No difference in beta-diversity (between all dietary treatments) was observed (P = 0.135).

The relative abundances of 76 genera were significantly different (P < 0.05; Bejamini-Hochberg FDR corrected) between NIT and CTL diets, 46 between COMB and CTL and 13 between MDDG and CTL–full details in S3 Table.

### Relationships with methane production

Differences in relative abundances of bacteria and archaea associated with methane emissions were assessed between diets. The relative abundance of *VadinCA11* was greater for the CTL dietary treatment relative to other dietary treatments (NIT and MDDG, P <0.001, COMB, P = 0.004). *Methanosphaera* and *Methanobrevibacter* were reduced for the NIT (P = 0.003; P = 0.017), no difference was observed in COMB dietary treatments (NS; NS), an numerical increased observed in MDDG dietary treatments (P = NS; NS). *Succinivibrio* was increased for the NIT (P = 0.018) and COMB (P = 0.051) dietary treatments, and remained at similar levels for the MDDG (NS; P = 0.470) dietary treatment. These data are summarized in Table 3.

**Table 3:** Mean relative abundance (standard deviation) as a fraction of total prokaryotic reads for each additive treatment (NIT, MDDG and COMB) and significance of genera associated with methane emissions for each diet group relative to the CTL diet. P-Value corrected using Benjamini-Hochberg FDR.

Taxonomy	CTL	NIT		COMB		MDDG	
	RA (SD)	RA (SD)	P-Value	RA (SD)	P-Value	RA (SD)	P-Value
*VadinCA11*	0.64 (0.18)	0.30 (0.10)	<0.001	0.40 (0.18)	0.004	0.27 (0.14)	<0.001
*Methanosphaera*	0.10 (0.06)	0.03 (0.03)	0.003	0.08 (0.05)	0.473	0.16 (0.08)	0.139
*Methanobrevibacter*	6.84 (3.45)	4.13 (2.22)	0.017	5.37 (2.38)	0.333	9.76 (5.71)	0.327
*Succinivibrio*	0.14 (0.11)	0.33 (0.21)	0.018	0.29 (0.21)	0.051	0.10 (0.10)	0.470

### Relationships with feed efficiency

**Fig 3:**
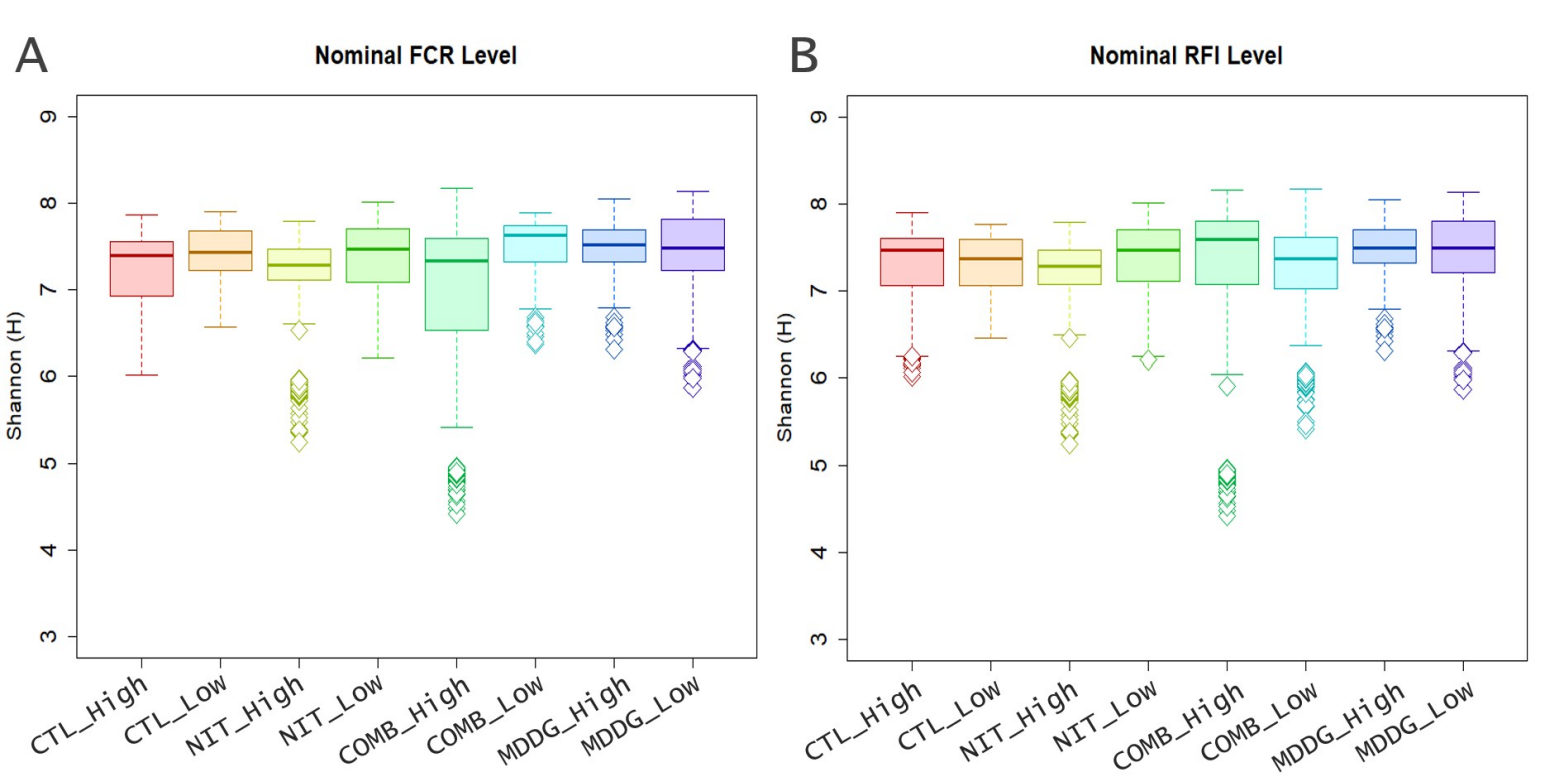
Differences in diversity using the Shannon index (H).

Overall microbial populations were associated with both nominal FCR (R^2^ = 0.187, P < 0.001) and RFI levels (R^2^ = 0.185, P <0.001). Firmicutes and Bacteroidetes are abundant taxa and their ratio (F:B ratio) differed between dietary treatments: 36.7:34.0 (1.190), 38.4:33.7 (1.360), 31.5:42.0 (0.843) and 35.6:33.5 (1.213) for CTL, MDDG, NIT and COMB diets respectively. Although there were some significant relationships between F:B ratio and feed efficiency for small sub-sets of the data (e.g. RFI for AAx steers on the MDDG dietary treatment; r = 0.76) most were weak and non-significant. Nominal RFI and FCR levels (low vs. high) and Shannon indexes were compiled into box plots across each diet (Fig 3), however there were no clear differences between groups.

Taxa present at < 1% relative abundance were discarded and the remainder assessed for relationships with feed efficiency (using general linear regression, as described by McCann et al. [5]. Within the MDDG diet, the relative abundance of Actinobacteria was negatively related to FCR level (P = 0.028). No significant relationships between feed efficiency and phyla were detected for the CTL, NIT or COMB dietary treatments. At the family level and for the MDDG dietary treatment, FCR value (P = 0.034) was negatively related to the relative abundance of Veillonellaceae, RFI level (P = 0.025) was negatively related to Prevotellaceae and RFI value (P = 0.009) was positively related to Prevotellaceae. RFI value (P = 0.014) was related to positively related to Methanobacteriaceae and negatively related to Lachnospiraceae (P = 0.037) and Succinivibrionaceae (P = 0.026) within the MDDG dietary treatment. There were no significant relationships between feed efficiency and relative abundances of families for the CTL or NIT dietary treatments. Within the COMB dietary treatment Veillonellaceae was negatively related to FCR value (P = 0.017) and Fibrobacteraceae was positively related to RFI value (P = 0.019).

## Discussion

### Diet effects on the rumen microbiota

Previous studies have shown marked diet effects on rumen microbial communities [7, 15, 25] and these generally agrees with our results. Studies have also shown the ability of the rumen microbiota to be influenced by age and stage of growth [26]. Veneman et al. [15] did not detect clustering of bacterial communities (assessed using NMDS) when evaluating effects of linseed oil or nitrate supplementation; however, there was slight clustering of archaeal communities. Similar results were also reported by Popova et al. [16] when assessing the effects of linseed and nitrate supplementation.

The increases in relative abundances of *Fibrobacter* and *Ruminococcus* for the NIT dietary treatment agrees with Veneman et al. [15], however in contrast to their study, *Pseudobutyrivibrio* abundances decreased. *Veillonella* has been identified as a denitrifier [27, 28] and others have noted an increase in response to nitrate feeding [28, 29], whilst others reported non-significant differences in relative abundances of *Veillonella parvula Veillonella dispar* [27]. However, in the present study *Veillonella* was more abundant in samples from the CTL diet in comparison with both nitrate-supplemented diets. The reason for this difference may be linked to different experimental methodologies as two of the previous studies [28, 29] used culture-based techniques which may result in a different picture if there are varying proportions of unculturable bacteria in comparison to our *in vivo* study. An increase in relative abundances of *Succinivibrio* were observed in NIT and COMB dietary treatments (relative to CTL and MDDG), indicating the potential of these taxa for nitrate reduction. Granja-Salcedo et al. [30] also reported increases in relative abundances of *Succinivirbio* and decrease in *Methanobrevibacter* in Nellore steers supplemented with encapsulated nitrate.

Poulsen et al. [19] showed that the reduction in methane emissions in response to rapeseed oil supplementation was related to reductions in the relative abundance of Thermoplasmata (Methanomassiliicoccaceae) and increases in *Methanosphaera* and *Methanobrevibacter*. Under their conditions, the relative abundance of Thermoplasmata levels seemed to explain more variation in methane emissions than the previously studied Methanobacteriales (*Methanosphaera* and *Methanobrevibacter*). However, the relative abundance of these groups of methanogens does not explain all variation in methane emissions–for example Danielsson et al. [31] found that numbers of unclassified *Methanomassiliicoccaceae* were 1.5 fold higher in low methane emitting dairy cows compared to high emitting dairy cows. However it must be noted that substantially lower methanogen abundances were reported by Poulsen et al. [19] compared to the current study. More recently, Eger et al. [32] saw a reduction in *Methanobrevibacter* species in response to a dietary additive used to reduce methane emissions (*in vitro*). Responses may depend on the basal diet and consequent relative abundances of these methylotrophic and hydrogenotrophic methanogens. It seems likely that effects on methane emissions in the current study represent a combination of effects with the lipid increasing Methanobacteriales from 6.7% to 10.3%, but decreasing the more methanogenic Thermoplasmata (*VadinCA11*) from 0.6% to 0.3%. The increase in Thermoplasmata (*VadinCA11*) in samples from the CTL dietary treatment in the current study may be explained by the presence of rapeseed meal in the CTL diet. Rapeseed meal, which was present only in the CTL diet, contains precursors (Glucosinolates and sinapine) for trimethylamine [33] which is a substrate utilized by *VadinCA11*.

### Relationship with feed efficiency

In the current study, there were no overall relationships between relative abundances of numerically important taxa and feed efficiency, however a few significant relationships for relatively minor taxa within certain dietary treatments were observed. This is not surprising given the well documented ability of microbial populations to adapt to dietary changes, which may have masked differences in feed efficiency influenced by changes in microbial communities [34, 35].

The absence of large and consistent relationships between microbial abundances and feed efficiency, as well as sporadic relationships with the relative abundance of individual taxa, is in general agreement with previous studies. Those studies often sampled extremes of low and high feed efficiency, in contrast to the current approach of sampling the full range of feed efficiencies. Rius et al. [36] found that PCA scores based on relative abundances of rumen microbial populations were not able to distinguish low and high RFI dairy cows. Similarly, Myer et al. [37, 38] also found that UniFrac PCoA plots of microbial communities were not able to distinguish low and high ADFI and ADG steers, whilst McGovern et al. [11] found no difference in community diversity between low (efficient) and high (inefficient) RFI bulls.

Although no clustering was observed, Rius et al. [36] did report that abundances of Fibrobacteraceae and Prevotellaceae were higher in less efficient cattle, whilst Lachnospiraceae were more abundant in more efficient animals. Jewell et al. [10] reported that inefficient dairy cows had increased levels of *Anaerovibrio*, *Clostridiales*, *Prevotella* and Ruminococcaceae, however other OTUs within each taxon were also more abundant in efficient animals. As seen in this study, differences in relative abundances relating to feed efficiency are not consistent across dietary treatments. Relationships with individual taxa have not been consistent across experiments [39].

Firmicutes to Bacteroidetes (F:B) ratio has been previously associated with feed efficiency in cattle [40, 41], as well as in humans and mice [42]. This ratio was highest in samples from the MDDG diet, but there were no significant relationships between F:B and feed efficiency measures for any diet. Ramirez et al. [41] found that dried distillers grains with solubles (lipid based additive to the diet) significantly reduced the ratio. However, the findings of Ramirez et al. [41] are not in agreement with the current study in which there were no significant effect of lipid on F:B ratio, this is possibly due to the higher dietary ether extract levels in Ramirez study (58 g/kg DM compared to 36.7 g/kg DM in this study). McGovern et al. [11] found that F:B ratios did not differ between efficient and inefficient animals.

In the present study, steers fed diets with nitrate inclusion had higher FCR values compared to steers offered the CTL diet (P < 0.05), although no significant difference was observed in RFI between the diets. Ley et al. [43] showed that obese mice had fewer Bacteroidetes compared to lean mice, which the authors suggested to be the result of more effective release of energy through digestion. However, an increased relative abundance of Bacteroidetes in samples from the NIT was noted in this study. A recent study by Shabat et al. [6] found that more efficient animals have a less diverse rumen microbial community. These authors suggest that this decreased diversity allows for more relevant metabolites to be produced and thus, more substrates made available for the host animal to use.

A lower number of OTUs was observed in samples from the MDDG diet when assessed using rarefaction plots, however no significant difference in Shannon diversity index estimates was observed. This is in agreement with Veneman et al. [15], who found no significant differences in Shannon diversity, for either bacteria or archaea, when comparing nitrate, linseed oil and control diet treatments. In the present study, significant differences in Shannon diversities were seen between LIMx and AAx steers associated with RFI within the NIT diet, with lower diversity observed for LIMx steers. LIMx are more efficient than AAx [14, 44], which supports the findings of Shabat et al. [6] who demonstrated lower diversity in more efficient Holstein cows. However there were no differences between feed efficiencies (either RFI or FCR) and rumen microbial diversity using the Shannon index for the CTL, MDDG or COMB diets. Myer et al. [37, 38] reported no differences in Shannon diversities of either rumen or jejunal microbiota between efficient and inefficient animals.

## Conclusion

The observation of occasional, but inconsistent, relationships between microbial abundances and feed efficiency suggests that there may be more complex and as yet unidentified mechanisms involved. Differences in metabolite production may be more or less related to changes in the microbiota depending on other factors such as basal diet and animal type.

The rumen microbiota was influenced by dietary lipid and nitrate supplementation, with four taxa *Methanomassiliicoccales*, *Methanobrevibacter*, *Methanosphaera*, and *Succcinivibrio* being, at least by one diet, significantly affected. Relative abundances of Succinivibrio were increased, whilst Methanobrevibacter decreased, in NIT and COMB dietary treatments (relative to CTL and MDDG) indicating the nitrate reducing potential of the genus Succinivbrio.

## Supporting information

S1 Fig Classification of animals into low (L) or high (H) RFI groups. (TIFF)

S2 Fig Rarefaction plot showing observed species for CTL, NIT, MDDG and COMB dietary treatments. (TIFF)

S1 Table Relative abundances of taxa from phylum to genus, mapping file and primer details. (XLSX)

S2 Table Core and unique microbiota between dietary treatments. (XLSX)

S3 Table Comparison of genera in dietary treatments (NIT, MDDG and COMB) relative to control (CTL). (XLSX)
