# Supplementary figures and images for: Links between the rumen microbiota, methane emissions and feed efficiency of finishing steers offered dietary lipid and nitrate supplementation
Source: PLoS One. 2020 Apr 24;15(4):e0231759. doi: 10.1371/journal.pone.0231759 (PMC7182223; doi:10.1371/journal.pone.0231759)

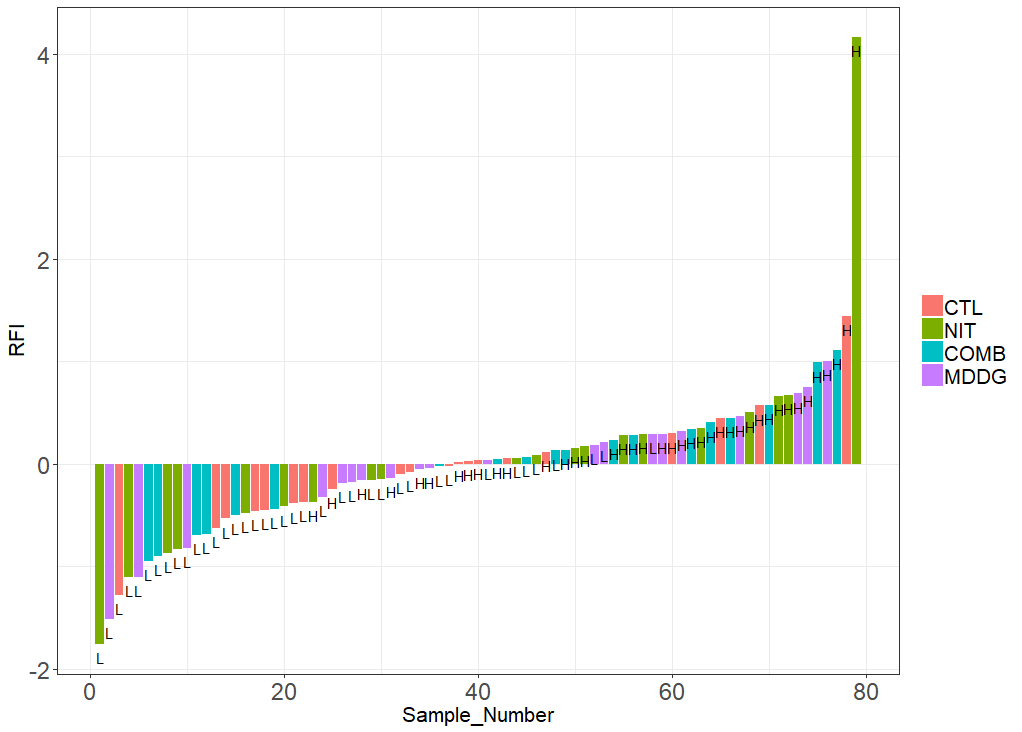

Supplement: S1 Fig — (TIFF) [file pone.0231759.s001.tiff]

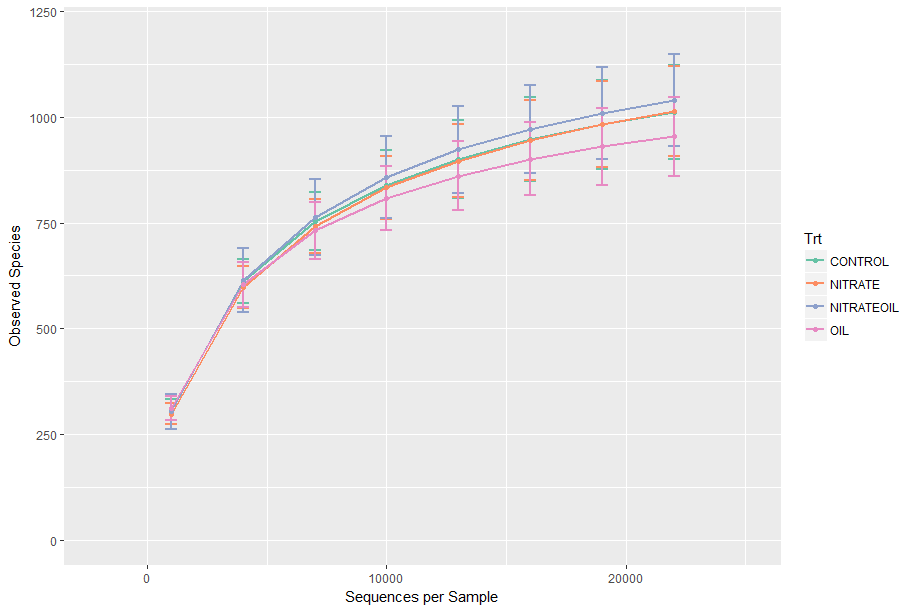

Supplement: S2 Fig — (TIFF) [file pone.0231759.s002.tiff]
